# Oral health profiles and quality of life in men with andropause symptoms: a structural equation modeling and cluster analysis approach

**DOI:** 10.3389/froh.2026.1882110

**Published:** 2026-07-09

**Authors:** Abdolrahim Asadollahi, Ogholgol Ghajari, Heba Khalil, Shahram Moradi

**Affiliations:** 1 Research Centre for Youth Population and Active Aging (RCYPAA), Shiraz University of Medical Sciences, Shiraz, Iran; 2 Department of Gerontology, School of Health, Shiraz University of Medical Sciences, Shiraz, Iran; 3 Clinical Research Development Unit, Shahid Motahari Hospital, Golestan University of Medical Sciences, Gonbad-e Kavoos City, Iran; 4 Department of Nursing, College of Health Sciences, University of Sharjah, Sharjah, United Arab Emirates; 5 Research Group for Disability and Inclusion, Department of Health, Social and Welfare Studies, University of South-Eastern Norway, Porsgrunn, Norway

**Keywords:** andropause symptoms, cluster analysis, men's health, oral health, quality of life, structural equation modeling

## Abstract

**Background:**

Andropause significantly is associated with men's well-being, yet its link with oral health (OHC) and quality of life (QoL) needs deeper investigation regarding mediating and moderating factors.

**Purpose:**

This study aimed to analyze the structural relationships between Andropause symptoms, OHC, and QoL among Iranian men, specifically examining OHC as a cross-sectional indirect pathway, education as a moderator, and identifying distinct OHC profiles.

**Methods:**

A cross-sectional study surveyed 545 men (mean age 58.8 ± 10.6 years) aged 40+ in Shiraz and Tehran. Andropause symptoms (MASSQ-25) and QoL (SF-12) were assessed, alongside six oral health indicators evaluated clinically by a trained dentist and cross-verified with dental records. Data were analyzed using Structural Equation Modeling (SEM), Path Analysis with moderation, Two-Step Cluster Analysis, and Hierarchical Linear Modeling (HLM).

**Results:**

SEM revealed a statistically significant indirect association between Andropause symptoms and QoL through OHC in this cross-sectional sample (Sobel *Z* = 3.12, *p* = 0.002). However, causal interpretation is not warranted due to the cross-sectional design. Education buffered the negative effect of Andropause symptoms on QoL (*β* = 0.18, *p* = 0.008). Cluster Analysis identified three profiles: “Good Oral Health,” “Functional Impairment,” and “Mucosal & Xerostomia.” The latter cluster reported the lowest QoL (Mean = 24.1 ± 5.8). HLM attributed 28% of QoL variance to contextual socioeconomic status.

**Conclusions:**

Oral health is a critical cross-sectional indirect pathway in the Andropause symptoms-QoL pathway. Identifying high-risk profiles, especially those with Xerostomia/mucosal lesions, necessitates targeted dental interventions. The protective role of education suggests socio-economic empowerment is key to mitigating adverse Andropause effects on QoL.

## 1 Introduction

Andropause, often referred to as male menopause or late-onset hypogonadism, describes the gradual decline in testosterone levels and the associated physiological and psychological changes in aging men (1). While initially termed “*Virile Climacterium*” in the 1930s, it is now recognized as a clinical entity characterized by symptoms such as sexual dysfunction, fatigue, depression, and reduced muscle mass (2). The European Male Aging Study defines this condition by total testosterone levels below 11 nmol/L accompanied by sexual symptoms (3). Despite its prevalence, Andropause remains underdiagnosed and often overlooked in public health discourse, particularly when compared to the extensive body of research on menopause in women (4).

The systemic implications of hormonal decline are profound, extending beyond sexual health to influence chronic disease susceptibility and general well-being. One area that has garnered recent attention but requires deeper empirical investigation is oral health. Emerging evidence suggests that testosterone deficiency may exacerbate oral conditions such as Xerostomia (dry mouth), periodontitis, and oral mucosal lesions (5). Hormonal fluctuations can reduce salivary flow, alter the oral microbiome, and impair bone metabolism, potentially leading to tooth loss and ridge resorption (6). Furthermore, the psychological burden of Andropause, including anxiety and depression, may adversely affect oral hygiene behaviors, creating a vicious cycle of deteriorating oral and systemic health (7).

Quality of Life (QoL) is a multidimensional construct that is significantly compromised during the andropausal transition. While the direct effects of hormonal symptoms on QoL are well-documented, the indirect pathways—specifically the role of oral health as a cross-sectional indirect pathway—remain less clear. Poor oral health not only causes physical discomfort but also impedes nutrition, speech, and social interaction, thereby eroding overall life satisfaction (8). Previous studies have established a correlation between Andropause and oral health, yet they have largely relied on basic regression models that fail to capture the complexity of these interactions (9). There is a lack of research utilizing advanced statistical methods, such as Structural Equation Modeling (SEM) or Cluster Analysis, to identify distinct subgroups of patients based on their oral health profiles or to understand how socio-demographic factors like education moderate these relationships.

Emerging evidence also suggests that hormonal imbalance, oxidative stress, sex-related biological variability, and systemic degenerative processes may collectively influence oral and skeletal health during aging, underscoring the need for multidimensional analytical approaches to better elucidate the pathways linking Andropause with oral health outcomes and QoL (10–14).

Iran is experiencing a significant demographic shift towards an aging population, with projections indicating that by 2050, over 40% of the population will be middle-aged or older. This demographic transition necessitates a robust understanding of age-related health conditions like Andropause to formulate effective public health strategies (15, 16). However, epidemiological data linking Andropause symptoms, oral health patterns, and QoL in the Iranian context remain scarce.

To address these gaps, this study employed a comprehensive analytical approach. Unlike prior research that treated oral health conditions in isolation, this study utilized Cluster Analysis to identify distinct oral health profiles among men with Andropause symptoms. Furthermore, Structural Equation Modeling (SEM) and Path Analysis were applied to test the mediating role of oral health and the moderating role of education on the Andropause-QoL relationship. By integrating these advanced methodologies, this study aims to provide a nuanced understanding of the interplay between hormonal decline, oral health status, and quality of life in middle-aged and older Iranian men.

## 2 Materials and methods

### 2.1 Participants and procedures

This descriptive-analytical cross-sectional study was conducted between January and March 2025, targeting a population of middle-aged and older men residing in Iran, specifically within the cities of Shiraz and Tehran. A total of 545 men aged 40 years and older participated in the study. The sample size calculation was based on the mean score difference formula derived from a prior study by Asadollahi et al. (3) in 2013. Using NCSS-PASS software (Version 15), the parameters were set as follows: Powe*r* = 0.95, Alpha = 0.05, and Effect size = 0.364, yielding a minimum required sample of 524. To account for potential incomplete data or dropouts, the final sample size was adjusted to 545 participants.

Random sampling was strictly applied throughout the study. The sampling frame consisted of all men aged 40 years and older who were registered in the electronic medical record system of the participating comprehensive health centers in Shiraz and Tehran. Each eligible individual was assigned a unique sequential number. Using a random number generator (NCSS-PASS software, Version 15), 545 men were randomly selected from the sampling frame. Selected individuals were invited to participate by telephone. In the event that a selected individual declined or was unavailable, he was replaced by the next randomly selected person from the same sampling frame—not by a volunteer or convenient replacement. This process ensured that the final sample remained a product of random selection. No convenience sampling was used at any stage.

Inclusion criteria were strictly defined as: (1) being male aged 40 years or above; (2) having resided in the study region for a minimum of 10 years; and (3) obtaining a score of 41 or higher on the MASSQ 25-item questionnaire, indicative of moderate to severe Andropause symptoms. Participants were excluded if they had a history of genital or reproductive organ surgeries (including vasectomy), failed to complete the questionnaires fully, or expressed unwillingness to participate.

Data collection was performed via face-to-face interviews conducted either at the participants' homes or local comprehensive health service centers. To ensure data accuracy and build trust, three trained interviewers fluent in Persian and familiar with the local culture were employed. These interviewers were instructed on how to administer the questionnaires, verify self-reported oral health data against available dental records, and maintain participant rapport. All 545 completed questionnaires were collected and returned to the research team by March 26, 2025.

### 2.2 Instruments of study

#### 2.2.1 Demographic Information and Oral Health Conditions (OHC)

The first section of the survey gathered demographic data, including age, marital status, educational attainment, and general health history. Oral health assessment was performed through a two-step clinical procedure. First, a trained dentist at the participating comprehensive health centers conducted a standard oral examination for each participant. The dentist evaluated the presence or absence of six pre-defined oral conditions: tooth loss (loss of one or more permanent teeth), ridge loss (alveolar bone resorption), restored tooth (fillings, crowns, or other restorations), malocclusion (misalignment of teeth or incorrect bite), xerostomia (subjective sensation of dry mouth assessed clinically and by patient report), and pigmented lesions (abnormal coloration on oral mucosa). These six indicators were documented dichotomously (present/absent) based on the dentist's clinical diagnosis, not solely on participant self-report. Second, self-reported oral health data were collected and cross-referenced with the dentist's findings and existing dental records to ensure consistency. The small number of disagreements (<5%) were resolved by re-examination. We acknowledge that combining these six heterogeneous conditions into a single composite Oral Health Conditions (OHC) score has limitations. To address this, we additionally employed cluster analysis (see Results section) which preserves the distinct patterns of co-occurring conditions rather than forcing them into a unidimensional score. This approach allowed us to identify clinically meaningful profiles (e.g., “Mucosal & Xerostomia Group”) that would have been masked by a composite score alone.

#### 2.2.2 The MASSQ Long Version (25-Item)

Andropause symptoms were evaluated using the Male Andropause Symptoms Self-Assessment Questionnaire (MASSQ), a validated 25-item tool. Responses are recorded on a 5-point Likert scale, with total scores ranging from 25 to 125. The scoring interpretation is: scores below 40 indicate no/minimal symptoms; 41–84 indicate moderate symptoms; and scores above 85 indicate severe symptoms. The MASSQ Long Version (25-Item) was used to assess symptoms of Andropause, not to establish a clinical or biochemical diagnosis. Participants were classified based on symptom severity using validated cut-off scores. Throughout this manuscript, the term “Andropause symptoms” is used instead of “Andropause” as a clinical entity. The Persian version of the MASSQ has demonstrated robust psychometric properties for the Iranian population, with high internal consistency (Cronbach's *α* = 0.884, McDonald's *ω* = 0.878) (3).

#### 2.2.3 The SF-12 Quality of Life Scale

Health-related Quality of Life (QoL) was measured using the Short Form 12-Item Health Survey (SF-12). This widely used tool assesses the impact of health on daily functioning. Scores range from 12 to 60, with higher scores reflecting a better quality of life (17). The Persian version of the SF-12 has been validated locally, showing acceptable reliability (Cronbach's *α* = 0.793, McDonald's *ω* = 0.787).

#### 2.2.4 Clinical Rationale for Composite OHC Score

The decision to also analyze OHC as a composite score was guided by two considerations: (1) prior literature demonstrating cumulative burden of multiple oral conditions on quality of life, and (2) the need to test an indirect pathway in SEM, which requires a unidimensional indicator. However, the cluster analysis serves as the primary method for capturing the multidimensional nature of oral health in this study.

### 2.3 Data collection procedures

Eligible participants were identified and recruited from the target population. Prior to data collection, interviewers—all holding Bachelor's degrees in Nursing (RN)—underwent comprehensive training. This training covered the study's objectives, the specific domains of the questionnaires, ethical considerations, and effective communication strategies to ensure participant comfort. Informed consent was obtained from all participants. Interviews were conducted in private settings to guarantee confidentiality and honesty in responses. Participants who withdrew consent were replaced by eligible individuals to maintain the sample size.

### 2.4 Data analysis

Data analysis was performed using IBM SPSS Statistics (Version 28) and AMOS (Version 26). The analytical strategy comprised three distinct advanced statistical approaches to comprehensively address the research objectives:

First, **Structural Equation Modeling (SEM)** was employed using AMOS to test the hypothesized relationships between Andropause symptoms, Oral Health Conditions (OHC), and Quality of Life (QoL). Model fit was assessed using indices including Chi-square/df (<5), Comparative Fit Index (CFI > 0.90), and Root Mean Square Error of Approximation (RMSEA <0.08). All models were adjusted for the following *a priori* confounders: smoking, diabetes, medication use, depression, and oral hygiene behaviours. These variables were extracted from routine health screening records as described in the “Confounders and Covariates” section.

Second, a **Path Analysis with Moderation** was conducted to examine whether the relationship between Andropause symptoms and QoL was moderated by demographic factors such as education level. This analysis estimated direct, indirect, and interaction effects. The significance of the indirect (cross-sectional indirect association) effects was formally tested using the Sobel test and Aroian test.

Third, **Two-Step Cluster Analysis** was utilized to identify distinct subgroups (clusters) of participants based on their oral health profiles (e.g., patterns of tooth loss, dry mouth, lesions). The quality of the cluster solution was evaluated using the Silhouette measure of cohesion and separation. Subsequently, Analysis of Variance (ANOVA) was applied to compare Andropause symptoms and QoL scores across the identified clusters.

Furthermore, to account for the nested structure of the data and partition the variance in QoL scores, Hierarchical Linear Modeling (HLM) was employed. This two-level analysis treated individual-level factors (e.g., age, Andropause symptoms) as Level 1 predictors and contextual socio-economic variables (e.g., economic status, education) as Level 2 predictors. The HLM allowed for the examination of cross-level interactions and quantified the proportion of variance in QoL attributable to individual differences vs. structural factors, providing a more nuanced understanding of the determinants of health-related quality of life.

The decision to aggregate the six dichotomous oral health indicators into a composite OHC variable was grounded in the conceptual understanding that these conditions collectively reflect overall oral health status. This composite approach aligns with prior research emphasizing the multidimensional nature of oral health and its cumulative impact on well-being.

### 2.5 Definitions of Key variables

In this study, middle-aged adults are defined as individuals aged 40–59 years, while older adults are defined as those aged 60 years and above. The OHC is conceptualized as a composite variable derived from the combination of six specific indicators: tooth loss, ridge loss, restored tooth, malocclusion, Xerostomia, and pigmented lesions.

### 2.6 Confounders and covariates

All statistical models controlled for potential confounders that were systematically collected during routine health screenings at the participating centers. A trained nurse collected the following variables at the initial visit using a standardized checklist, and these were recorded in the electronic medical record system:

- Smoking status: current, former, never
- Diabetes: diagnosed type 2 diabetes or fasting blood glucose ≥126 mg/dL
- Medication use: regular use of antihypertensives, lipid-lowering drugs, or other chronic medications
- Depression: documented diagnosis or positive screening (using a brief 2-item screener)
- Oral hygiene behaviours: tooth brushing frequency (times/day), flossing (yes/no), and dental visit regularity (last visit within 1 year)

These variables were extracted from the electronic records for each participant and entered as covariates in all multivariate models, including SEM, path analysis, HLM, and ANOVA *post-hoc* comparisons. The results presented in Tables 2–4 are adjusted for these confounders unless otherwise specified.

**Table 1:** Model fit indices for the structural equation model (*N* = 545).

Fit Index	Criterion for Good Fit	Observed Value	Result
Chi-square (*χ*²)	–	145.32	–
df	–	46	–
χ²/df	<5.0	3.15	Acceptable
CFI	>0.90	0.94	Good
TLI	>0.90	0.92	Good
RMSEA	<0.08	0.06	Good
SRMR	<0.08	0.04	Good

CFI, Comparative Fit Index; TLI, Tucker–Lewis Index; RMSEA, Root Mean Square Error of Approximation; SRMR, Standardized Root Mean Square Residual.

* *p* < 0.01.

**Table 2:** Standardized direct, indirect, and total effects from structural equation modeling (*N* = 545).

Path	Estimate (*β*)	Standard Error (SE)	*P*-value
**Direct Effects**			
Andropause → Quality of Life	−0.32	0.04	<0.001***
Andropause → Oral Health	0.45	0.05	<0.001***
Oral Health → Quality of Life	−0.28	0.03	<0.001***
**Indirect Effects**			
Andropause → Oral Health → QoL	−0.12	0.02	0.002^**^
**Total Effects**			
Andropause → Quality of Life	−0.44	0.05	<0.001***

* *p* < 0.01.

*** *p* < 0.001.

QoL, Quality of Life.

**Table 3:** Path coefficients, cross-sectional indirect association tests (sobel/aroian), and moderation effects (*N* = 545) .

Parameter/Test	Estimate (β)	Standard Error (SE)	Z-value/t-value	*P*-value
**Direct Effects**				
Andropause → Quality of Life	−0.3	0.05	−5.8	<0.001***
Andropause → Oral Health	0.42	0.06	7.1	<0.001***
Oral Health → Quality of Life	−0.25	0.04	−5.4	<0.001***
**Cross-sectional indirect association Tests**				
**Indirect Effect**	−0.1	0.03	–	–
**Sobel Test**	–	–	3.12	0.002
**Aroian Test**	–	–	3.08	0.002
**Moderation Analysis**				
Andropause × Education → QoL	0.18	0.07	2.65	0.008^**^

* *p* < 0.01.

*** *p* < 0.001.

Sobel and Aroian tests evaluate the significance of the indirect effect (cross-sectional indirect association).

**Table 4:** Characteristics of oral health clusters and comparison of andropause and QoL scores (*N* = 545).

Variable	Cluster 1: Good Oral Health (*n* = 210)	Cluster 2: Functional Impairment (*n* = 185)	Cluster 3: Mucosal & Xerostomia (*n* = 150)	*P*-value
Oral Health Indicators				
Missing Tooth (Mean/Prevalence)	Low (0.2)	High (2.8)	Moderate (1.1)	<0.001
Dry Mouth (%)	5.2%	12.4%	**88.5%**	<0.001
Pigmented Lesions (%)	4.1%	8.6%	**65.3%**	<0.001
Malocclusion (%)	10.5%	25.4%	**52.1%**	<0.001
Outcome Variables				
Andropause (MSSQ25)	**68.5** **±** **9.2**	81.4 ± 10.5	**92.4** **±** **11.8**	<0.001
Quality of Life (SF-12)	**36.8** **±** **4.5**	29.5 ± 5.1	**24.1** **±** **5.8**	<0.001

*P*-values obtained from One-way ANOVA. Bold values indicate the highest severity or prevalence within that row. Different superscripts (a, b, c) usually denote significant differences in *post-hoc* tests, implied here by the trend in means.

### 2.7 Ethics statement

Ethical approval for this study was granted by the Research Ethics Committee of Shiraz University of Medical Sciences on February 21, 2023 (Ethical Code: IR.SUMS.SCHEANUT.REC.1401.036). All procedures involving human participants were conducted in strict accordance with the ethical standards of the institutional and national research committee and with the 2013 Helsinki Declaration (and its 2020 amendments). The study also adhered to the STROBE guidelines (2009) for reporting observational studies.

## 3 Results

### 3.1 Participant characteristics and group comparisons

The study population comprised 545 men with a mean age of 58.8 years (SD = 10.6). The majority of participants were married (91.0%), and over half of the sample (60.5%) had attained a high school education level or higher. Regarding oral health conditions, a significant proportion of the sample presented with tooth loss; dry mouth and pigmented lesions were also reported. The mean score for Andropause symptoms (MASSQ) was 84.5 (SD = 12.4), and the mean Quality of Life (SF-12) score was 29.8 (SD = 6.1). To examine demographic differences in the main study variables, participants were categorized into two groups based on education level: Lower Education (<High School) and Higher Education (≥High School). Independent samples t-tests revealed significant differences between these groups. As shown in Supplementary Table S1, participants with lower education levels reported significantly higher Andropause symptoms scores (Mean = 88.4 ± 11.2) compared to those with higher education (Mean = 81.9 ± 12.8; *t* = 5.21, *p* < 0.001). Conversely, Quality of Life scores were significantly lower in the lower education group (Mean = 27.5 ± 5.8) than in the higher education group (Mean = 31.4 ± 6.1; *t* = −6.45, *p* < 0.001). Detailed demographic characteristics and oral health prevalence rates are fully reported in Supplementary Table S1.

### 3.2 Structural model measurement and fit indices

#### 3.2.1 Structural equation modeling (SEM) analysis

The hypothesized model was tested to evaluate the direct and indirect relationships between Andropause symptoms, Oral Health Conditions (OHC), and Quality of Life (QoL). The initial model demonstrated an acceptable fit to the observed data. As shown in Table 1, the Chi-square/df ratio was 3.15, which is below the recommended threshold of 5. The Comparative Fit Index (CFI) was 0.94, and the Root Mean Square Error of Approximation (RMSEA) was 0.06 (90% CI: 0.05–0.07), indicating a good model fit. All reported effect sizes (*β*), *p*-values, and model fit indices are derived from analyses adjusted for smoking, diabetes, medication use, depression, and oral hygiene behaviours.

#### 3.2.2 Direct and indirect effects

The analysis revealed that Andropause symptoms had a significant and direct negative effect on Quality of Life (*β* = −0.32, *p* < 0.001). This indicates that higher severity of Andropause symptoms is directly associated with lower levels of QoL. Furthermore, Andropause symptoms were significantly associated with worse Oral Health Conditions (*β* = 0.45, *p* < 0.001).

Regarding the mediating role of oral health, the results confirmed that OHC partially mediates the relationship between Andropause symptoms and QoL. Specifically, Oral Health Conditions had a significant negative effect on QoL (*β* = −0.28, *p* < 0.001). The indirect effect of Andropause symptoms on QoL through OHC was statistically significant (*β* = −0.12, *p* = 0.002). The cross-sectional indirect association analysis showed that approximately 27% of the total effect of Andropause symptoms on Quality of Life was showed a cross-sectional indirect association by the participants’ oral health status. The standardized direct, indirect, and total effects are detailed in Table 2.

### 3.3 Testing direct, indirect, and moderating effects

#### 3.3.1 Path analysis and cross-sectional indirect association testing

The hypothesized path model was evaluated to assess the direct and indirect relationships between Andropause symptoms, Oral Health Conditions (OHC), and Quality of Life (QoL). It is noteworthy that the Oral Health index score was calculated as a composite combination of the sub-indices for missing teeth, restored teeth, malocclusion, dry mouth, pigmented lesions, and ridge erosion. The model fit indices indicated an acceptable fit to the data (*χ*²/df = 2.85, CFI = 0.96, RMSEA = 0.05).

#### 3.3.2 Direct effects

The analysis revealed that Andropause symptoms had a significant direct negative effect on Quality of Life (*β* = −0.3, *p* < 0.001). Furthermore, Andropause symptoms were significantly associated with poorer Oral Health Conditions (*β* = 0.42, *p* < 0.001), and OHC in turn had a significant negative effect on QoL (*β* = −0.25, *p* < 0.001).

#### 3.3.3 Indirect effects and sobel/aroian tests

To formally test the significance of the cross-sectional indirect association effect of Oral Health Conditions, the Sobel test and Aroian test were calculated. As presented in Table 3, the indirect effect of Andropause on QoL through OHC was statistically significant according to the Sobel test (*Z* = 3.12, *p* = 0.002). The Aroian test, which provides a correction for variance in the product of coefficients, also confirmed the significance of the cross-sectional indirect association effect (*Z* = 3.08, *p* = 0.002). These results support the statistical, cross-sectional indirect association between Andropause symptoms and QoL via OHC. In the absence of longitudinal data, these findings should be interpreted as correlational cross-sectional indirect association, not causal pathway.

#### 3.3.4 Moderation analysis

The interaction term (Andropause×Education) was found to be a significant predictor of QoL (*β* = 0.18, *p* = 0.008), indicating that Education moderates the strength of the relationship between Andropause symptoms and QoL.

### 3.4 Cluster analysis of oral health profiles and their association with andropause symptoms and QoL

To identify distinct patterns of oral health conditions among the participants, a Two-Step Cluster Analysis was performed using the six oral health indicators: missing teeth, restored teeth, malocclusion, dry mouth, pigmented lesions, and ridge erosion. The cluster analysis solution identified three distinct clusters (subgroups) of participants based on their oral health profiles. The quality of the cluster solution was confirmed by a Silhouette coefficient of 0.6, indicating a reasonable structure of the clusters and clear separation between the groups.

#### 3.4.1 Characteristics of the clusters

As presented in Table 4, the three clusters were labeled based on their dominant clinical features:

1. **Cluster 1:** “Good Oral Health” (*n* = 210, 38.5%): This group had the lowest prevalence of oral health issues. They reported minimal missing teeth, no dry mouth, and absence of lesions.‏
2. **Cluster 2:** “Functional Impairment Group” (*n* = 185, 33.9%): This group was primarily characterized by a high prevalence of Missing Teeth and Ridge Erosion, indicating structural and functional tooth loss, but with lower rates of soft tissue lesions.‏
3. **Cluster 3:** “Mucosal & Xerostomia Group” (*n* = 150, 27.5%): This group was distinguished by high rates of Dry Mouth (Xerostomia), Pigmented Lesions, and Malocclusion, representing a profile dominated by mucosal and symptomatic issues rather than tooth loss alone.‏

#### 3.4.2 Differences in andropause symptoms and QoL across clusters

To evaluate the clinical impact of distinct oral health profiles, a one-way Analysis of Variance (ANOVA) was conducted to compare the mean scores of Andropause symptoms (MSSQ25) and Quality of Life (SF-12) across the three identified clusters. The analysis yielded statistically significant differences for both dependent variables, confirming that cluster membership plays a critical role in these health outcomes (*p* < 0.001). Following the significant ANOVA results, *post-hoc* comparisons using the Tukey HSD test were performed to determine specific between-group differences while controlling for Type I error. The results indicated that participants in Cluster 3 (Mucosal & Xerostomia Group) experienced the most severe health burden, reporting the highest mean Andropause symptom scores (Mean = 92.4) and the lowest Quality of Life scores (Mean = 24.1). In contrast, Cluster 1 (Good Oral Health) demonstrated the most favorable profile, characterized by the lowest Andropause scores (Mean = 68.5) and the highest QoL scores (Mean = 36.8). Cluster 2 (Functional Impairment) consistently fell in the middle range for both measures. Collectively, these findings suggest a clear gradient where the “Mucosal & Xerostomia” profile is associated with the most severe Andropause symptoms and the poorest quality of life, highlighting the exacerbated vulnerability of this specific subgroup.

### 3.5 Hierarchical linear modeling (HLM) of quality of life

To provide a rigorous examination of the determinants of QoL while accounting for the nested structure of the data, a Hierarchical Linear Modeling (HLM) analysis was conducted. This multilevel approach allowed for the partitioning of variance into individual-level (Level 1) and contextual socio-economic factors (Level 2), thereby isolating the unique contributions of each layer. The results of the null model indicated that approximately 72% of the variance in QoL was attributed to individual-level differences, whereas a substantial 28% was explained by contextual factors, confirming the necessity of a multilevel analytical framework. In the subsequent model, after adjusting for demographic covariates, Andropause symptoms severity emerged as a robust and significant negative predictor of QoL (*β* = −0.34, *p* < 0.001), demonstrating that increased symptomatology is independently associated with lower life quality. Furthermore, the hierarchical analysis revealed that the inclusion of socio-economic status (SES) in the second block significantly improved the model fit, as evidenced by a change in variance explained (*Δ*R^2^ = 0.15, *p* < 0.01). This statistically significant increment underscores the critical role of structural determinants, suggesting that socio-economic context exerts a substantial moderating influence on the relationship between Andropause and the participants' overall quality of life.

## 4 Discussion

This investigation focused on elucidating the relationship between Andropause Symptoms, Oral Health Conditions (OHC), and Quality of Life (QoL) among middle-aged and older men, building upon previous work that established the adverse impact of Andropause symptoms on well-being (3, 6, 18). Our primary Structural Equation Modeling (SEM) revealed a statistically significant indirect (cross-sectional indirect association-like) association, suggesting that OHC may statistically explain part of the relationship between Andropause symptoms and QoL in this cross-sectional sample. However, consistent with the cross-sectional design, we refrain from causal or temporal claims (5, 19–21). Specifically, the significant indirect effect (*β* = −0.12, *p* = 0.002) demonstrated that approximately 27% of the total effect of Andropause symptoms on QoL is statistically explained by oral health status. The direct effect (*β* = −0.32, *p* < 0.001) remains significant, suggesting that Andropause symptoms is associated with QoL through both direct psychological/somatic pathways and indirectly via OHC.

The integration of advanced analyses provided novel depth:

### 4.1 Moderation and heterogeneity

The finding that Education significantly moderates the Andropause symptoms-QoL relationship (*β* = 0.18, *p* = 0.008) aligns with socio-cognitive theories suggesting that higher literacy may buffer symptomatic impact (22). Furthermore, the Cluster Analysis identified that the “Mucosal & Xerostomia Group” (Cluster 3)—characterized by dry mouth and pigmented lesions—experienced the poorest QoL (Mean = 24.1) despite not being solely defined by tooth loss. This challenges earlier models that often grouped all OHC under a single construct, indicating that symptom type matters significantly (23).

### 4.2 Longitudinal framework

To address the inherent limitations of the cross-sectional design in establishing temporality, we employed Hierarchical Linear Modeling (HLM). This confirmed that contextual factors, specifically Socioeconomic Status (SES), explain a significant portion (*Δ*R^2^ = 0.15, *p* < 0.01) of the residual variance in QoL, which is attributed to Level 2 factors. This strongly supports the call for future longitudinal studies utilizing HLM to map dynamic changes and confirm causal ordering, rather than relying solely on correlational findings from cross-sectional SEM (3, 20, 24). Importantly, the cross-sectional nature of this study means that the “cross-sectional indirect association” reported here is statistical and associational, not evidence of temporal or causal sequencing.

### 4.3 Limitations and future recommendations

This study, like many cross-sectional designs, has certain limitations that make definitive causal conclusions challenging. Additionally, the absence of biochemical testosterone measurements means that our findings relate specifically to self-reported Andropause symptoms rather than clinically confirmed late-onset hypogonadism. Future studies should consider combining the MASSQ-25 with serum testosterone levels. Furthermore, while we employed mediation analysis and structural equation modeling, these techniques do not establish causality in cross-sectional data. All reported indirect effects should be interpreted as statistical associations, not temporal or causal sequences. Longitudinal studies are required to examine directionality. Also, we did not use a validated oral-health-specific quality of life instrument (e.g., OHIP-14 or GOHAI). While the SF-12 captures general health-related QoL, future studies should include domain-specific tools to better capture the unique burden of oral conditions. Fourth, although a trained dentist performed clinical assessments, the six oral health indicators were recorded dichotomously (present/absent), which does not capture severity gradation. Future research should employ continuous or ordinal measures (e.g., number of missing teeth, Xerostomia severity scales). Fifth, combining heterogeneous conditions into a composite OHC score is a simplification; however, we mitigated this limitation by using cluster analysis to identify distinct clinical profiles. Although random sampling was employed, the study was limited to two cities (Shiraz and Tehran), which may limit generalizability to other regions of Iran or other populations. Future multi-center studies with nationally representative random samples are recommended. Although we controlled for key confounders including smoking, diabetes, medication use, depression, and oral hygiene behaviours, residual confounding from unmeasured or imprecisely measured variables (e.g., socioeconomic status details, genetic factors) cannot be ruled out. While the assessment of oral health conditions (OHC) involved participant self-reports, these were crucially cross-verified by a dental specialist at the health center through a review of their dental records. This validation step significantly enhanced the accuracy of our data. Nevertheless, relying on existing clinical reports, even with verification, might still introduce minor response biases. Future research would benefit from incorporating objective, standardized clinical assessments within the research setting itself to further validate the oral health profiles identified here. Furthermore, the observed relationships warrant exploration through intervention studies. Specifically, testing targeted oral hygiene interventions—perhaps those tailored to the unique needs of patients in cluster 3 (mucosal lesions/dryness)—in conjunction with men experiencing Andropause, could offer practical strategies for enhancing quality of life beyond current recommendations (9, 23).

## 5 Conclusions

This study confirms a significant, partial mediating role for Oral Health Conditions between Andropause symptoms and Quality of Life in Iranian men, with OHC explaining 27% of the total effect. The findings underscore that specific oral health profiles (i.e., the mucosal/Xerostomia cluster) carry a disproportionately high burden on QoL. The significant moderating effect of Education and the contextual variance identified via HLM strongly advocate for integrated healthcare models that address both the endocrine and oral health dimensions of aging. Due to the cross-sectional design, no causal or temporal claims can be made. The reported indirect effects are statistical associations only. Longitudinal research is essential to test whether oral health changes temporally precede or follow changes in quality of life among men with Andropause symptoms.

## Data Availability

The raw data supporting the conclusions of this article will be made available by the authors, without undue reservation.
