# Supplementary material for: Oral health profiles and quality of life in men with andropause symptoms: a structural equation modeling and cluster analysis approach
Source: Front Oral Health. 2026 Jul 9;7:1882110. doi: 10.3389/froh.2026.1882110 (PMC13392986; doi:10.3389/froh.2026.1882110)
Supplement: Supplementary file 1 [file Table1.docx]

**Supplementary Table 1. Demographic and Clinical Characteristics of Participants by Education Level (N = 545)**

| **Variable** | **Total (N=545)** | **Lower Education (< High School) (n=215)** | **Higher Education (≥ High School) (n=330)** | **Test Statistic (t / χ²)** | **P-value** |
| --- | --- | --- | --- | --- | --- |
| **Age (Mean ± SD)** | 58.8 ± 10.6 | 59.2 ± 11.1 | 58.5 ± 10.2 | 0.711 | 0.478 |
| **Marital Status (n, %)** |  |  |  |  |  |
| Married | 496 (91.0%) | 190 (88.4%) | 306 (92.7%) | 2.61* | 0.106 |
| Single / Divorced / Widowed | 49 (9.0%) | 25 (11.6%) | 24 (7.3%) |  |  |
| **Clinical Scores (Mean ± SD)** |  |  |  |  |  |
| Andropause (MASSQ) | 84.5 ± 12.4 | **88.4 ± 11.2** | **81.9 ± 12.8** | 5.21 | **< 0.001*** |
| Quality of Life (SF-12) | 29.8 ± 6.1 | **27.5 ± 5.8** | **31.4 ± 6.0** | -6.45 | **< 0.001*** |
| **Oral Health Conditions (n, %)** |  |  |  |  |  |
| Missing Tooth | 312 (57.2%) | 135 (62.8%) | 177 (53.6%) | 4.12* | 0.042* |
| Dry Mouth (Xerostomia) | 298 (54.7%) | 125 (58.1%) | 173 (52.4%) | 1.71* | 0.191 |
| Pigmented Lesions | 210 (38.5%) | 95 (44.2%) | 115 (34.8%) | 4.85* | 0.028* |
| Malocclusion | 180 (33.0%) | 78 (36.3%) | 102 (30.9%) | 1.71* | 0.191 |
| Restored Tooth | 165 (30.3%) | 60 (27.9%) | 105 (31.8%) | 0.91* | 0.340 |
| Ridge Erosion | 95 (17.4%) | 45 (20.9%) | 50 (15.2%) | 2.71* | 0.100 |
| ***Note****: Continuous variables analyzed using Independent Samples t-test. Categorical variables analyzed using Chi-square (χ²) test. Bold values indicate statistical significance. ***p < 0.001, *p < 0.05.* | | | | | |
